# Synthesis and Characteristics of a pH-Sensitive Sol-Gel Transition Colloid for Coal Fire Extinguishing

**DOI:** 10.3390/gels9010069

**Published:** 2023-01-14

**Authors:** Yiru Wang, Qinglin Zheng, Hetao Su, Zijun Huang, Gengyu Wang

**Affiliations:** 1 School of Engineering and Technology, China University of Geosciences, Beijing 100083, China; 2 Key Laboratory of Deep Geodrilling Technology, Ministry of Natural Resources, China University of Geosciences, Beijing 100083, China; 3 School of Reliability and Systems Engineering, Beihang University, Beijing 100191, China

**Keywords:** coal fire extinguishing, pH-sensitive, sol-gel transition, fluidity, oxygen isolation

## Abstract

Coal fires, most of which are triggered by the spontaneous combustion of coal, cause a huge waste of resources and release poisonous and harmful substances into the environment, seriously threatening the safety of industrial production. Gel flame retardant plays a core role in coal fire prevention and extinguishing. Most gel flame retardants used in coal fires possess good sealing and oxygen isolation properties, but it is difficult for them to flow deep into fire areas due to their low fluidity. Some fire extinguishing agents with good fluidity lack leak-blocking performance. In order to simultaneously improve the fluidity, leakage sealing, and oxygen isolation effects of coal fire extinguishing colloids, a novel, pH-sensitive, sol-gel transition colloid was prepared using low methoxyl pectin (LMP), calcium bentonite (Ca-Bt), sodium bentonite (Na-Bt), and water as the main components. When the initial sol-state colloid absorbed acid gas products from coal combustion, the pH value decreased and a large amount of Ca^2+^ in Ca-Bt precipitated, thus immediately growing calcium bridges with LMP molecules that formed a three-dimensional network structure for gelation. The optimum ratio of the new colloid was determined through X-ray diffraction, tube inversion, shock shear-temperature scanning, and genetic algorithm. By testing the fire extinguishing performance of the colloid, the findings proved that the product had good oxygen isolation performance, strong adhesion ability, high thermal stability, and strong inhibition effects on coal combustion.

## 1. Introduction

Coal is the second largest petrochemical resource in the world and has always occupied an important position in the global energy consumption structure, with huge output and consumption. In 2021, 8.173 billion tons of coal were produced all over the world, with China topping the list [1]. Meanwhile, 86.17 exajoule of coal was consumed in China, accounting for more than half of the global total. China is one of the largest coal-producing and -consuming countries in the world, and also one of the regions with the most serious coal fires mainly triggered by spontaneous combustion of coal [2,3,4,5,6,7]. Coal fires cause a huge waste of resources and the release of sulfur dioxide, hydrogen sulfide, carbon monoxide, and other gases, which damages the environment [8]. Meanwhile coal fires pose a serious threat to safe industrial production [9], causing a large number of casualties and equipment damage, as well as derivative disasters, such as gas and coal dust explosion, roof collapse, water intrusion, etc. Since coal fires are difficult to find and extinguish which are deeply buried, cover a large area, burn at high temperatures, and can re-ignite, coal fires are a worldwide problem [10,11,12,13]. Researchers have proposed a variety of fire extinguishing technologies and countermeasures. Due to advantages such as covering the residual coal in the goaf and filling the voids of coal bodies, gel has excellent fire extinguishing performances in cooling, sealing, and water retention, and has been gradually regarded as a core agent of coal fire extinguishing [14].

An efficient and environmentally friendly colloid flame retardant has been considered as an important coal fire extinguishing technology [15,16]. The traditional inorganic silicon gel has the key advantages of low cost, high fluidity, and rapid gelation, and it is widely used in coal fire extinguishing. However, silicon gel used in coal fires is not ideal because it does not retain water well, accompanied by cracking and crushing of the consolidated body after water loss that releases harmful gases. Ren et al. [17] prepared a novel sodium silicate/polymer composite gel with anionic polyacrylamide that effectively resolved the common water-loss cracking and crushing problems of inorganic consolidated silica gels. Many researchers have investigated foam gels. Compared with pure foam, foam gel has the advantages of high stability, superior temperature resistance, and fluidity [2,18]. Qiao et al. [19] prepared a high-foaming gel foam without a foaming agent to improve the stability time and foaming ratio, while Xue et al. [20] proposed a gel-stabilized foam with a higher viscosity of the foaming solution. The foam gels they studied had excellent fire extinguishing capacities. Lu et al. [21] proposed a new type of antioxidant gel foam with procyanidins in the gel system, while Xue et al. [22] prepared a temperature-sensitive microencapsulated fire-retardant foam gel, both of which had good inhibitory effects. The purpose of other researchers has been to optimize new gel materials by polymerizing or compounding organics in order to improve specific characteristics. Cheng et al. [23,24] prepared intelligent gels by polymerizing organics to achieve good thermal stability and high toughness. Li et al. [25,26] proposed fire extinguishing gels that used methylcellulose as a basic material to achieve high water absorption and temperature sensitivity. Han et al. [27] prepared a new biomass gel foam based on sodium alginate, calcium L- lactate, alkyl glycoside, and tea saponin in order to create an environmentally friendly fire prevention and extinguishing material for coal mines. The above new fire extinguishing gels achieved thermal stability, strong inhibition, high water retention, and resilience and could prevent re-ignition after fire extinguishing, thus overcoming the limitations of traditional gels. In practical engineering applications, the fluidity, sealing, and oxygen isolation properties of the gels should also be considered.

**Figure 1:**
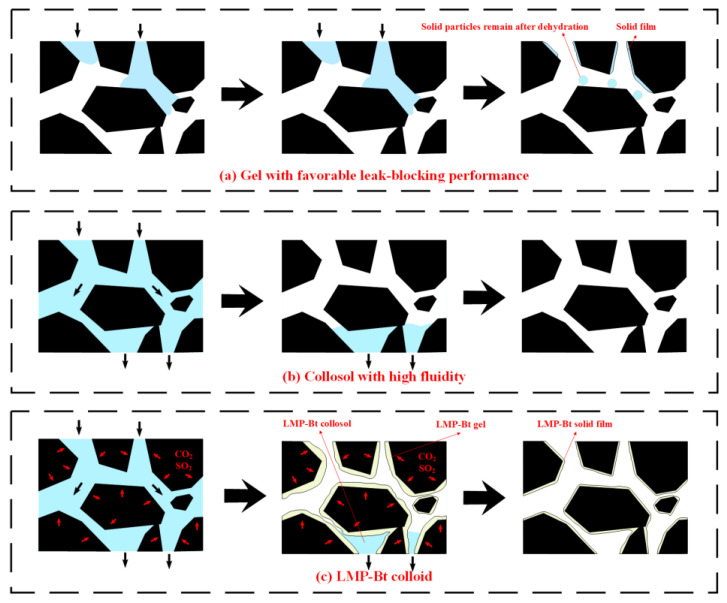
Fluidity and oxygen isolation of colloid: (**a**) Gel with favorable leak-blocking performance, (**b**) Collosol with high fluidity and (**c**) LMP-Bt colloid.

The gel flame retardant used in coal fires fills the cracks between coal bodies to occupy and block air flow channels. This filling method provides good sealing and oxygen isolation, but a colloid with reduced fluidity has difficulty penetrating deep into the fire area, as shown in Figure 1a. Thus, the fire cannot be effectively extinguished. Figure 1b shows that a colloid with high fluidity has difficulty remaining in the channels or cracks between coal bodies for a long time, which leads to a decline in sealing and oxygen isolation. Therefore, a colloid with high fluidity and oxygen isolation ability needs to be produced.

As shown in Figure 1c, a sol-gel transition colloid is a novel solution for coal fire extinguishing since it has a high pH value and good fluidity in the initial state. When injected deep into the coal combustion zone, CO_2_, SO_2_, and other acid gases caused by coal combustion are rapidly absorbed by the colloid layer contacting the coal surface. This process leads to a decrease in the pH value of the colloid layer, thereby reducing its fluidity. At this time, the colloid layer is adhered to the coal surface, forming a gelatinous membrane that achieves oxygen isolation. Meanwhile, the pH value of the colloid not in contact with the coal body will not rapidly decrease, thus the colloid remains sol-like and continues to flow deep into the fire area. Therefore, the colloid features high fluidity, oxygen isolation, and gas product absorption. Low methoxyl pectin (LMP), selected as a component of the pH-sensitive sol-gel transition colloid, is a natural polymer compound formed by polymerization of galacturonic acid. It is widely found in terrestrial plants, and it is an easily available and low-cost material that can be mixed to increase colloidal consistency [28,29]. LMP colloid will change its flow state in response to the change in Ca^2+^ content [30]. When the content of Ca^2+^ is low or absent, the colloids are collosols. When the Ca^2+^ content increases, LMP molecules are crosslinked to form gels [31,32,33]. This unique ‘smart colloid’ may be an effective solution that simultaneously improves the fluidity of conventional colloids while maintaining good oxygen isolation capability. Bentonite (Bt) is a kind of clay-like mineral with a layered structure. Bt, with a cationic interlayer of calcium ions (Ca^2+^), is called calcium-based Bt (Ca-Bt), which responds to changes in pH values. When the pH value is high, Ca^2+^ between the Ca-Bt layers will be stable [34,35,36]. When the pH value decreases, Ca^2+^ will precipitate. When Ca-Bt is combined with LMP, Ca-Bt can provide LMP with the Ca^2+^ needed for sol-gel transition by sensing the change in pH value, which makes the application of LMP in coal fire extinguishing feasible.

The purpose of this study was to prepare a pH-sensitive, LMP/Bt intercalation colloid (LMP-Bt) to achieve sealing and oxygen isolation while ensuring good fluidity. In addition, the fire extinguishing performance was verified through various tests, including X-ray diffraction, orthogonal experiments, tube inversion, oscillatory shear tests with temperature scanning, weighing at constant temperature, thermogravimetric differential scanning calorimetry, etc. This paper provides theoretical and practical support for the synthesis and characteristics of the colloid.

## 2. Results and Discussion

### 2.1. LMP-Bt Colloid Microstructures

The layer spacing of Bt could be represented by the spacing of the {001} crystal plane. The layer spacing is an important index for determining whether a specified substance is inserted into the Bt layer. If the distance between the Bt layers is increased, this indicates that the specified substance had been inserted between the Bt layers. If the layer spacing is constant, this indicates that the specified substance is not inserted between the Bt layers [37].

**Figure 2:**
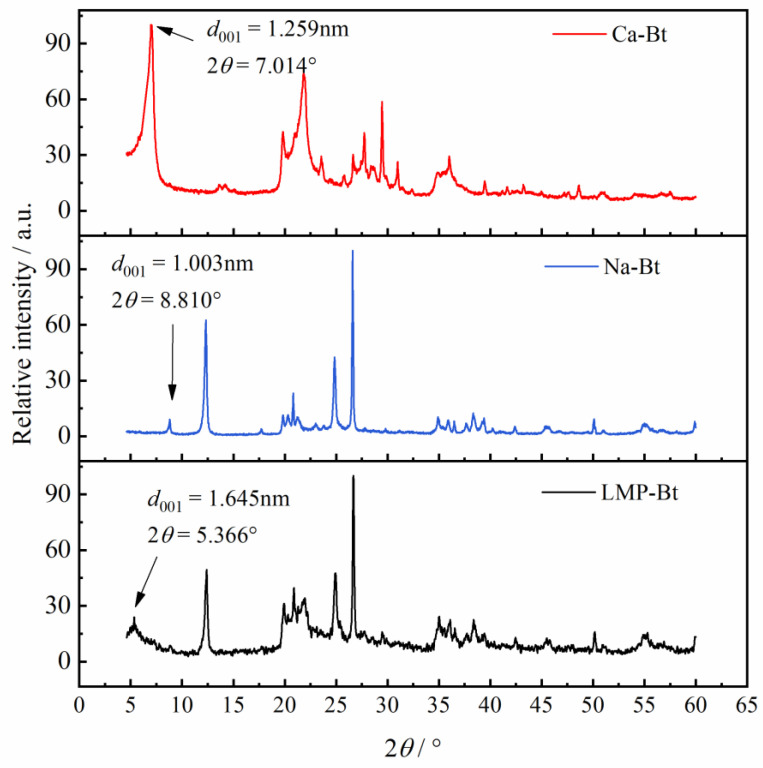
X-ray diffraction results of Ca-Bt gel, Na-Bt gel and LMP-Bt gel.

Figure 2 shows the X-ray diffraction results of the Ca-Bt, Na-Bt, and LMP-Bt gels. It could be seen that the {001} crystal planes of Ca-Bt and LMP-Bt corresponded to 2*θ* = 7.014° and 2*θ* = 5.366°, respectively. According to Bragg’s formula:

(1) 2dsinθ=nλ

where d is the spacing of the {001} crystal plane, θ is the corresponding angle of the {001} crystal plane, *n* is the reflection order (usually set as 1), and λ is the X-ray wavelength. Based on Equation (3), the spacings of the {001} crystal planes for Ca-Bt, Na-Bt, and LMP-Bt were 1.259, 1.003, and 1.645 nm, respectively. The layer spacing of LMP-Bt was significantly larger than that of Ca-Bt and Na-Bt, indicating that LMP was successfully inserted into the Bt layer to form an intercalation structure.

### 2.2. pH Sensitivity of LMP-Bt Colloid

#### 2.2.1. Mechanism for pH Sensitivity of LMP-Bt

**Figure 3:**
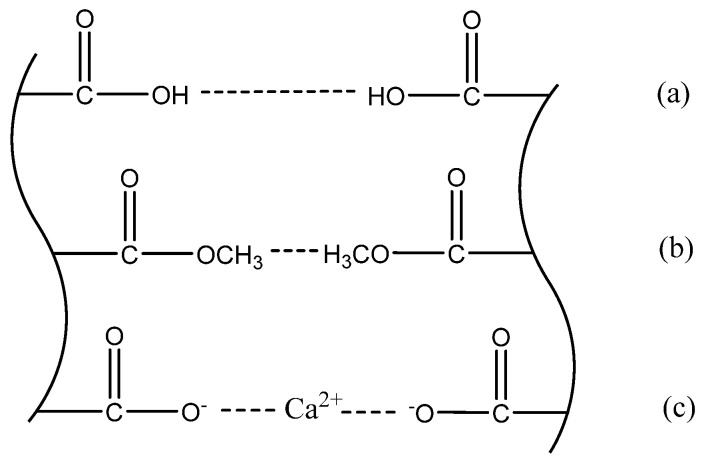
Eggshell model: (**a**) Hydrogen bond, (**b**) Water separating force between methoxy groups and (**c**) Calcium bridge.

LMP colloid is an intelligent colloid that can achieve sol-gel transition by sensing changes in external environmental factors, including pH value, Ca^2+^ content, etc. Some scholars have studied the gelation mechanism of LMP colloids, among which the ‘eggshell model’ is the widely accepted explanation of the gelation phenomenon of LMP colloid in the current field, as shown in Figure 3. When the three forces, including hydrogen bonding (a), the water separating force between methoxy groups (b), and calcium bridges (c) are small or absent, the LMP colloid is sol-like. When one or more of the forces reach a critical amount, the system will form a three-dimensional network structure called a gel in macroscopic expression.

**Figure 4:**
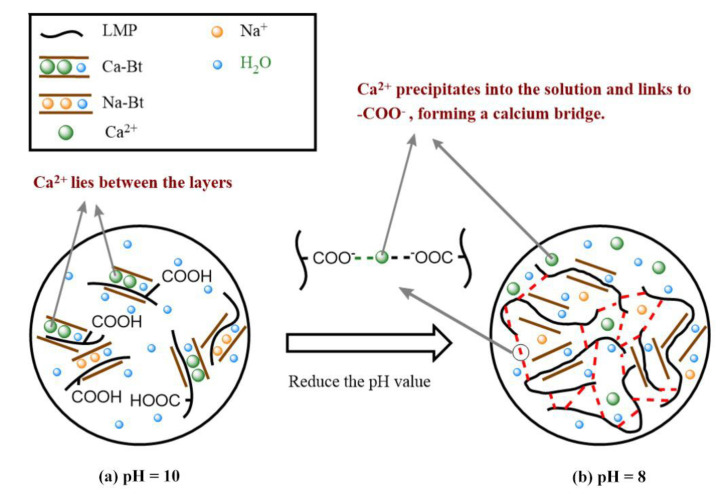
Gelation mechanism of the pH-sensitive LMP-Bt: (**a**) pH = 10 and (**b**) pH = 8.

The gelation mechanism of the LMP-Bt colloid is mainly related to the forces shown in Figure 3c. The initial pH value of the LMP-Bt colloid was approximately 10. Ca^2+^ in Ca-Bt will stably exist among its layers (Figure 4a) and will not enter the solution, so the forces do not appear. Due to the high pH value, the —COOH in LMP is ionized and the water separating force between methoxy groups is weak, thus the two forces in Figure 3a,b will not appear either. Therefore, the LMP-Bt is sol-like under these conditions. After the LMP-Bt colloid absorbs acid gas products from coal combustion, the pH value of the LMP-Bt will decrease, and Ca^2+^ in Ca-Bt will precipitate from the interlayer (Figure 4b) to the solution, immediately forming calcium bridges with LMP molecules, as shown in Figure 3c. When the amount of calcium bridges reaches a critical level, a three-dimensional network structure of the LMP-Bt will appear, macroscopically manifested as the formation of gel. When the pH value decreases to 8, sufficient Ca^2+^ precipitates from Ca-Bt for gelation. However, the pH value of Ca-Bt is still high, and the two forces in Figure 3a,b will not appear. Therefore, calcium bridges play a dominant role in the sol-gel transition of the LMP-Bt colloid.

#### 2.2.2. The Critical pH Value for Gelation

The experimental results regarding the critical pH value for gelation are shown in Table 1. The critical pH value for gelation was 6.5~8.0. Carbon dioxide, the main acidic gas product of coal combustion, dissolved in water to reduce the LMP-Bt pH value to 5.6. Therefore, the LMP-Bt colloid achieved sol-gel transition.

${\bar{k}}_{i,j}$ represents the average critical pH value of experimental samples and was calculated by Equation (2):

(2) $${\bar{k}}_{i,j}=\sum {\bar{k}}_{i,j}/a,\;i\in \left\{\mathrm{LMP},\;\mathrm{Ca}-\mathrm{Bt},\;\mathrm{Na}-\mathrm{Bt}\right\},\;j\in \left\{L-1,\;L-2,\;L-3\right\}$$

where *a* is the number of experiments with factor *i* and level *j*.

Table 2 shows the average critical pH values of the experimental samples for gelation.

The range of ${\bar{k}}_{\mathrm{Ca}-\mathrm{Bt},j}$ was the largest, at 1.08, indicating that the Ca-Bt content had the greatest effect on the critical pH value of colloid gelation. The range of ${\bar{k}}_{\mathrm{LMP},j}$ was 0.25. The range of ${\bar{k}}_{\mathrm{Na}-\mathrm{Bt},j}$ was the smallest, at only 0.08. The order of the effect of the three factors on the critical pH value of colloid gelation was ranked as follows: Ca-Bt content > LMP content > Na-Bt content. In addition, the higher the critical pH, the more likely the colloid was to undergo sol-gel transition in coal combustion areas, resulting in good oxygen isolation performance by easily attaching to the coal surface. Therefore, the critical pH value for gelation had a larger-the-better characteristic, and the optimal levels of components were: 12 g/L LMP, 40 g/L Ca-Bt, and 30 g/L Na-Bt.

An analysis of variance of the critical pH values for gelation was conducted, as shown in Table 3. 

The *p* value of the Ca-Bt content was less than 0.01, indicating that the Ca-Bt content had a very significant effect on the critical pH value. This is because Ca^2+^ was the main factor influencing the sol-gel transition of the LMP colloid at pH 6.5~8.0, and Ca-Bt was the only source of Ca^2+^. When the content of Ca-Bt increased, the total amount of precipitated Ca^2+^ increased at a critical pH value, providing enough Ca^2+^ to fully crosslink the LMP molecules and form the three-dimensional network structure.

The *p* value of the LMP content was greater than 0.05, indicating that the LMP content had no significant effect on the critical pH value. When Ca^2+^ entered the solution, two kinds of calcium bridges occured between LMP molecules: intermolecular and intramolecular calcium bridges. The intermolecular calcium bridges could promote gelation, while the intramolecular calcium bridges could not promote gelation. The appearance of the two calcium bridges was mainly determined by the content of LMP molecules. When the content of LMP molecules was low, intramolecular calcium bridges mainly appeared. With the increase in LMP molecules, intermolecular calcium bridges gradually appeared. Therefore, the critical pH value for gelation increased with increasing LMP content. However, when the content of LMP increased to a critical extent, the tendency of forming intramolecular calcium bridges became low. Although the proportion of intramolecular calcium bridges would still increase with increasing LMP content, it had little effect on the critical pH value. Therefore, there was no significant correlation between the critical pH value and the content of LMP due to relatively high content of LMP in our experiments.

Na-Bt also had no significant effect on the critical pH value. The main cation between Na-Bt layers is Na^+^. When the pH value decreases, Na^+^ will also precipitate to different degrees. Some studies [38,39] showed that although Na^+^ cannot crosslink with the —COO^-^ group of LMP to form a bridge like Ca^2+^, it can play a role in electrostatic shielding, reducing the electrostatic repulsion between LMP molecules and promoting the LMP molecules to get close to each other, thus forming hydrogen bonds between the —COOH groups, as shown in Figure 3a. This will promote gelation. However, when the pH value drops to 6.5~8.0, gelation can occur and most of the —COOH groups in LMP are ionized, unable to form hydrogen bonds, and existing in the form of —COO^-^. Even if Na^+^ precipitates, it cannot promote gelation. Therefore, the Na-Bt content had no significant effect on the critical pH value.

#### 2.2.3. Experimental Results of Gelation by Absorbing Gas Products of Coal Combustion

**Figure 5:**
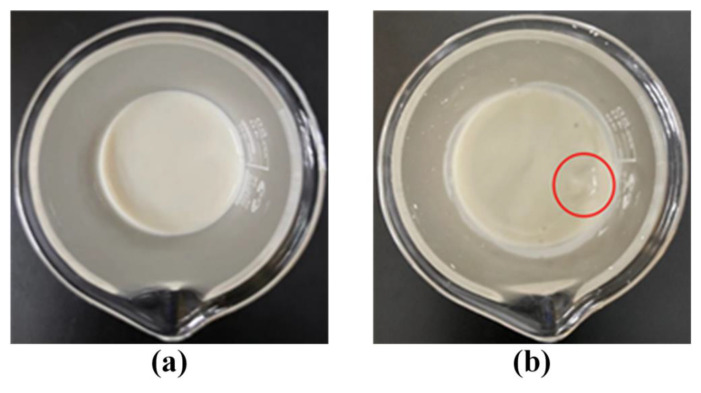
LMP-Bt colloid: (**a**) before absorbing gas products and (**b**) after absorbing gas products.

The surface state of the LMP-Bt colloid after absorbing gas products of coal combustion is shown in Figure 5. The LMP-Bt colloid presented as a collosol with a smooth surface before gas products were passed through. After absorbing the gas products, an obvious depression appeared on the surface of the LMP-Bt colloid around the insertion position of the gas outlet (red circle in Figure 5b). If this part of the colloid was still sol-like and fluid at this time, it would flow to the depression under the action of gravity and a horizontal surface would appear once again. If the depression remained stable for a long time, this indicated that this part of the colloid had achieved gelation. The colloid far away from the insertion position of the gas outlet retained a smooth surface, indicating that it remained in sol state. Thus, the LMP-Bt colloid directly in contact with gas products could be converted into a gel in a relatively short time, while other parts of the colloid remained in sol state.

### 2.3. Rheology of LMP-Bt Colloid

#### 2.3.1. Rheological Properties in Different pH Environments

The storage modulus (G’) refers to the energy stored by a material caused by its elastic deformation. Loss modulus (G”) refers to the energy loss of a material caused by its viscous deformation. When the value of G’ is greater than that of G”, the material tends to be solid. When the value of G’ is less than that of G”, the material tends to be liquid. When the value of G’ is close to that of G”, the material tends to be semi-solid.

**Figure 6:**
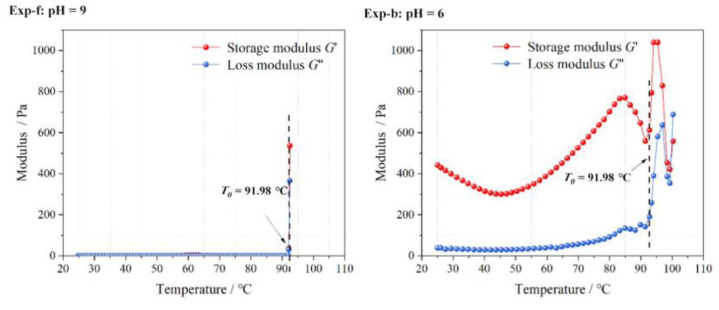
Elasticity modulus with increasing temperature at pH values of 6 and 9.

**Figure 7:**
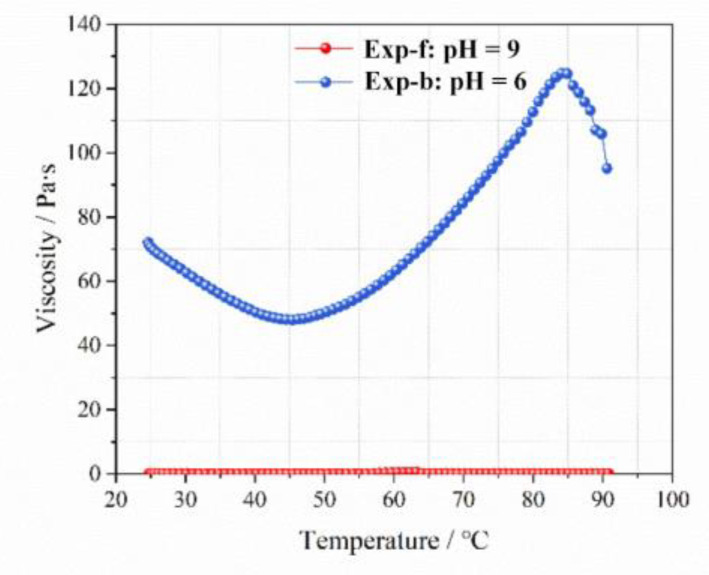
Viscosity with increasing temperature at pH values of 6 and 9.

Figure 6 and Figure 7 show the values of elastic modulus and viscosity of the LMP-Bt colloid with increasing temperature at pH values of 6 and 9, respectively. When the pH value was 9, the value of G’ was similar to that of G’’, with low viscosity. This indicated that the colloid was sol-like and had good fluidity. Thus, the colloid was able to reach deep into the fire area to achieve a good perfusion effect. When the pH value was 6, the value of G’ was greater than that of G”, and the viscosity was significantly greater than that of the colloid at the pH value of 9. This indicated that a colloid film isolating oxygen and inhibiting coal oxidation was formed due to gelatinous colloid staying on the coal surface for a long time.

When the temperature was low, slight changes occured in G’ and G”. When the temperature reached a specific value, both the values of G’ and G” produced large irregular vibrations due to the gradual loss of colloid moisture and gradual transformation from colloid to solid, leading to inaccurate results in the rheology tests. The modulus value was defined as the mutation point value, the ratio of the difference between which the maximum value to the maximum value was greater than 15%. The temperature corresponding to the mutation point was recorded as *T*_0_. Therefore in this paper, only the data before *T*_0_ are discussed.

#### 2.3.2. Rheological Properties at Different Ca-Bt Contents

**Figure 8:**
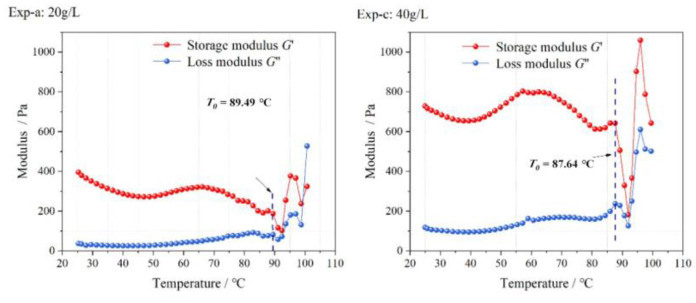
Elastic modulus with increasing temperature at different Ca-Bt contents.

The results in Section 2.2.2 showed that Na-Bt had no significant effect on the sol-gel transition of the LMP-Bt colloid. The contents of Ca-Bt and LMP were changed in the rheology tests. The elastic modulus and viscosity of the LMP-Bt colloid with different Ca-Bt contents was measured with the pH value fixed at 6. Figure 6 (Exp-b) and Figure 8 show the change in elastic modulus with increasing temperature at different Ca-Bt contents. When the pH value was 6, the three groups of LMP-Bt colloids, of which the G’ values were all greater than the G” values, were in gel state, indicating that the LMP-Bt colloids with a Ca-Bt content ranging from 20 to 40 g/L had no fluidity at the pH value of 6.

**Figure 9:**
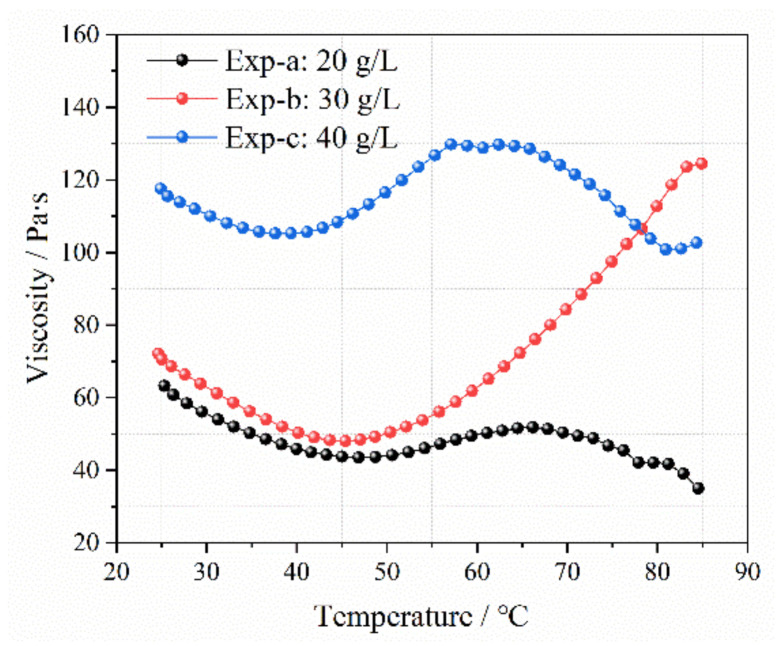
Viscosity with increasing temperature at different Ca-Bt contents.

Figure 9 shows the viscosity of the colloids at different Ca-Bt contents. At room temperature, the viscosity of the three groups of LMP-Bt colloids increased with increasing Ca-Bt content.

This was because the higher Ca-Bt content could provide more Ca^2+^, thus increasing the degree of LMP molecule crosslinking. However, due to the stronger molecular vibration, the calcium bridges between LMP molecules were gradually destroyed, and the colloid viscosity for the three groups decreased with the rise in temperature from 25 to 45 °C. When the temperature rose continuously from 45 to 65 °C, water evaporation accelerated and water loss increased the viscosity values. When the temperature exceeded 65 °C, the viscosity values of the colloids in Exp-a and Exp-c gradually decreased, while the viscosity value of the colloid in Exp-b gradually increased and exceeded that value in Exp-c at approximately 80 °C. For Exp-a, the Ca-Bt content was low and the degree of LMP molecule crosslinking was insufficient, so the thermal stability of the colloid was poor. For Exp-c, the Ca-Bt content was high, resulting in an excessive degree of crosslinking, which reduced the thermal stability of the colloid [31,32]. Therefore, when the temperature exceeded 65 °C, a mass of the calcium bridges in the colloids were destroyed in Exp-a and Exp-c, leading to a decline of viscosity. The colloid in Exp-b had the best Ca-Bt content, a moderate degree of crosslinking, and good thermal stability. As the temperature increased, the number of broken calcium bridges decreased, and the water continued to evaporate, so the colloid viscosity gradually increased.

The average viscosity was calculated by Equation (3).

(3) $$\bar{\eta }=\frac{\sum_{i=1}^{K}\eta_{i}}{K}$$

where $\bar{\eta }$ is the average viscosity value in Pa∙s; i is the serial number of viscosity value with temperature; $\eta_{i}$ is the viscosity value with a serial number of *i* in Pa∙s; and K is the number of viscosity values. As shown in Table 4, colloid viscosity was positively correlated with Ca-Bt content.

#### 2.3.3. Rheological Properties under Different LMP Content

**Figure 10:**
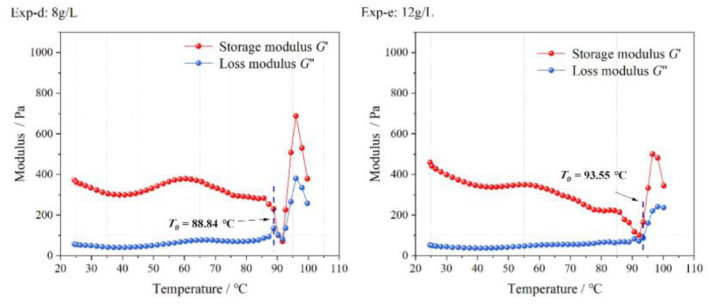
Colloid elastic modulus with increasing temperature at different contents of LMP.

Figure 2 (Exp-b) and Figure 10 show the change in elastic modulus of the LMP-Bt colloids with increasing temperature at LMP contents of 10, 8, and 12 g/L at the pH value of 6. The three groups of LMP-Bt colloids, of which the G’ values were all greater than the G” values, were in gel state, indicating that the LMP-Bt colloids with LMP contents ranging from 8 to 12 g/L had no fluidity at the pH value of 6.

**Figure 11:**
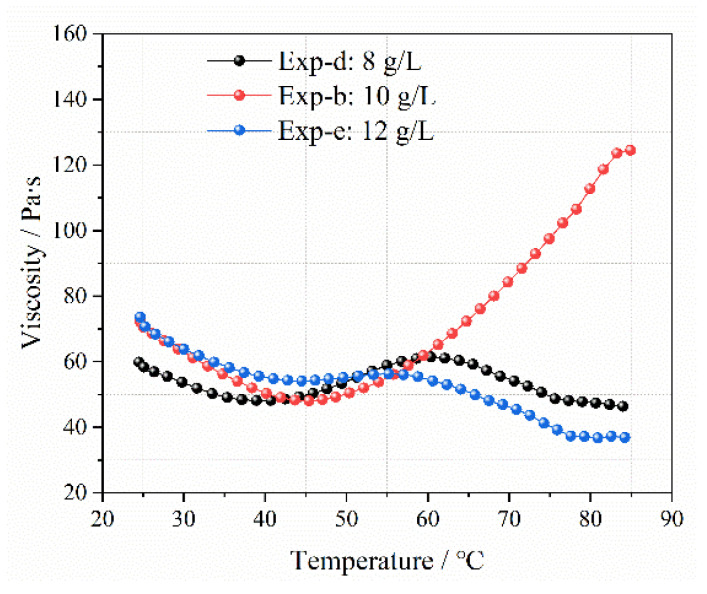
Colloid viscosity with increasing temperature at different contents of LMP.

Figure 11 shows the viscosity of the LMP-Bt colloids when the contents of LMP were 8, 10, and 12 g/L. 

The viscosity values of Exp-e were the greatest, followed by those of Exp-b and Exp-d. For Exp-d, due to the low content of LMP, the formation of effective calcium bridges was inhibited, which decreased the viscosity. With increasing LMP content, more effective calcium bridges appeared, so the colloid viscosity values in Exp-b were higher than those in Exp-d at the same temperature. When the LMP content continued to increase, the amount of LMP gradually became excessive but the Ca^2+^ content was insufficient, so the formation of effective calcium bridges slightly increased. This resulted in slightly higher viscosity values observed in Exp-e than in Exp-b at the same temperature. With the temperature increasing from 25 to 45 °C, the calcium bridges between LMP molecules were partially destroyed, and the viscosity values of the three groups of colloids decreased. When the temperature continued to rise from 45 to 60 °C, the viscosity values of the three groups of colloids increased due to water evaporation. When the temperature exceeded 60 ℃, the colloid viscosity values in Exp-d and Exp-e began to decline, while the values in Exp-b kept rising. For Exp-d, the thermal stability of the colloid was poor because of the low LMP content and the formation of few effective calcium bridges. For Exp-e, the thermal stability of the colloid was also poor due to insufficient Ca^2+^ content and weak degree of LMP molecule crosslinking. The average values of LMP-Bt colloid viscosity in Exp-d, Exp-b and Exp-e were calculated according to Equation (5). As shown in Table 5, the viscosity values of the colloid increased with increasing LMP content and then decreased.

### 2.4. Water Retention of LMP-Bt Colloid

In order to reduce the experimental error, the water retention rates of five groups of the same LMP-Bt gel samples were measured. Their average values were used to characterize the actual water retention rate of the LMP-Bt gel. Table 6 shows that the LMP-Bt gel retained approximately 59.53% moisture after constant temperature drying at 60 °C for 12 h.

### 2.5. Oxygen Isolation of LMP-Bt Colloid

Table 7 shows that the specific surface area of a coal sample without LMP-Bt colloid treatment was 7.56 m^2^/g, while that of a coal sample after LMP-Bt colloid treatment was 5.34 m^2^/g, a decrease of approximately 29.37%, indicating that the LMP-Bt colloid could still adhere to the coal surface in the form of a solid film after complete water loss to reduce the specific surface area of the coal. A smaller specific surface area of coal means that fewer active sites are exposed, which decreases the chance for oxygen to be adsorbed by the coal surface [40]. In some studies, silicates were used to fill the voids of coal and reduce the oxidation contact area of the coal, which inhibited the oxidation of coal. [26]. This was consistent with the findings of this paper. Therefore, the LMP-Bt colloid effectively reduced the adsorption capacity of coal for oxygen.

### 2.6. Adhesion of LMP-Bt Colloid

Table 8 shows the adhesion rates of coal + CMC-Na and coal + LMP-Bt (from Exp-9) samples. The average adhesion rate of the coal + CMC-Na samples was 16.14%, while that of the coal + LMP-Bt samples was 65.10%, indicating that the LMP-Bt colloid had good adhesion.

### 2.7. Thermal Stability of LMP-Bt Colloid

**Figure 12:**
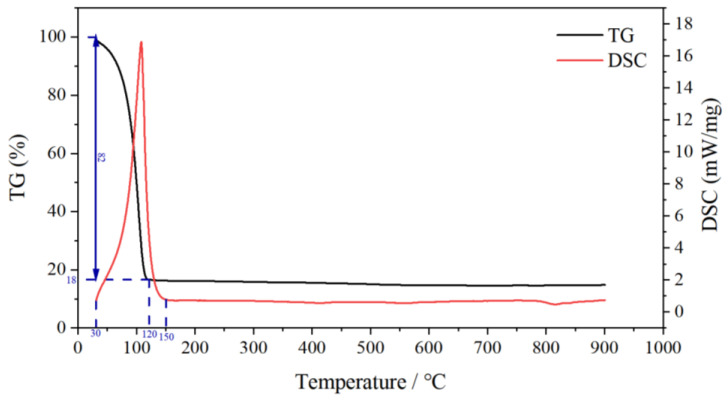
TG-DSC curves of the LMP-Bt gel.

Figure 12 shows the TG-DSC curves of the LMP-Bt gel. According to the TG curve, the mass of the LMP-Bt gel gradually decreased from 30 to 120 °C, which resulted from the gradual loss of water. After 120 ℃, the mass remained stable without significant change, indicating that the gel had lost water completely, and no mass loss was caused by component decomposition at 120~900 °C. According to the DSC curve, the LMP-Bt gel had an obvious endothermic peak at approximately 110 °C, which was caused by water evaporation, indicating that the LMP-Bt gel had a significant endothermic cooling effect. At 150~900 °C, there was no obvious exothermic peak, indicating that no exothermic decomposition reaction occurred at high temperature, suggesting that reignition of the gel would be difficult.

### 2.8. Inhibition of Coal Combustion

**Figure 13:**
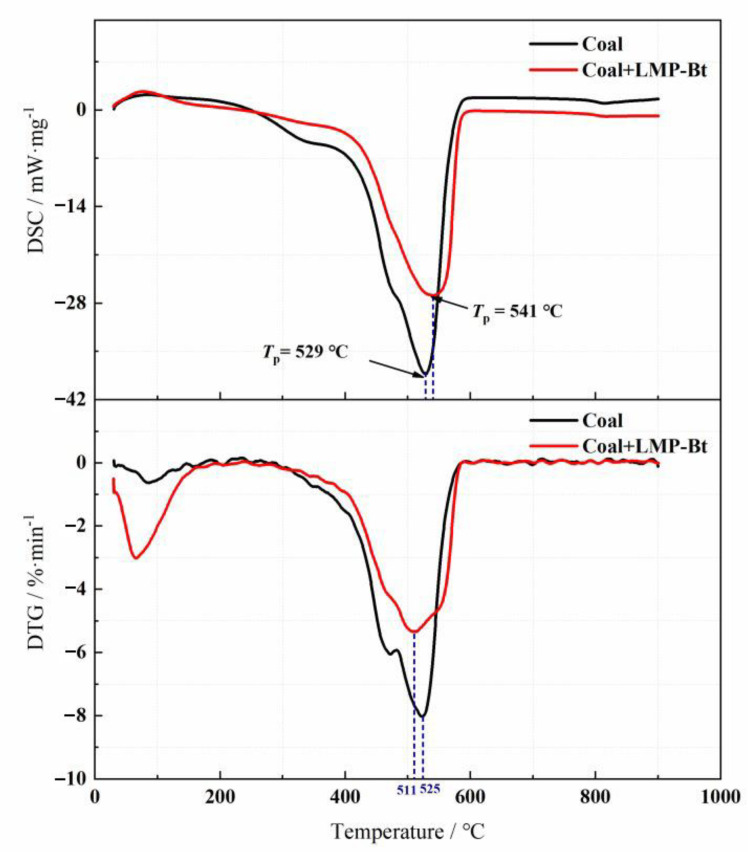
DTG-DSC curves of the coal and coal + LMP-Bt samples.

The DTG-DSC curves of the two samples (coal, coal + LMP-Bt) are shown in Figure 13. The DSC curve of the coal + LMP-Bt sample showed an endothermic peak at approximately 100 °C, indicating that the mass of the sample rapidly decreased at this time because of water evaporation. The variation trends of the DSC and DTG curves for the coal sample were the same as those for the coal + LMP-Bt sample, but the amplitude of variation was small for the coal + LMP-Bt sample. Zhou et al. [41] showed that after gel treatment, the weight loss rate of coal samples decreased and the released energy decreased. These results were consistent with the findings of the study. This was because the coal sample was at the evaporative desorption phase at approximately 100 ℃, thus the coal-oxygen composite reaction rate was accelerated and the primary gases (CO_2_, CH_4_, N_2_, etc.) in the coal pore began to desorb. The amount of gas desorption was greater than that of gas adsorption, resulting in mass loss. Meanwhile, a slight upward peak appeared in the DSC curve of the coal sample due to heat absorption during gas desorption.

**Figure 14:**
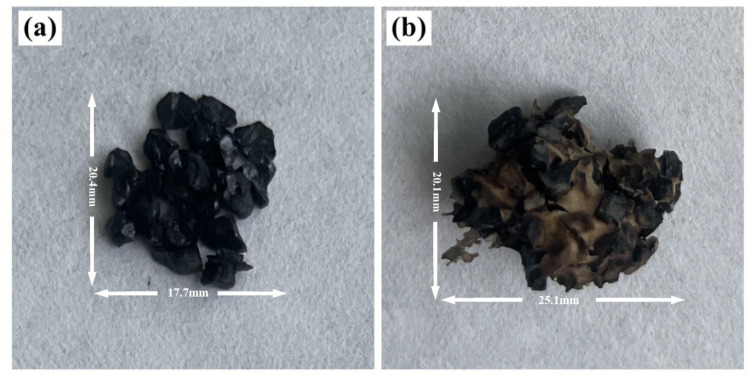
Partial samples at 300 °C for 1 h: (**a**) coal, (**b**) coal + LMP-Bt.

There were obvious exothermic peaks at 400~600 °C in each DSC curve, owing to the large amount of heat released from coal combustion. The absolute values of DTG for both samples were large in this temperature range due to the rapid reduction in coal mass. The exothermic peak for the coal sample appeared at approximately 529 °C, while the exothermic peak of the coal + LMP-Bt sample appeared at approximately 541 °C, indicating that the LMP-Bt colloid delayed the oxidation of coal. The exothermic peak area for the coal + LMP-Bt sample was significantly smaller than that for the coal sample, indicating that the exothermic heat of the coal sample was significantly reduced after LMP-Bt treatment. This was because the LMP-Bt colloid could still wrap the coal body to isolate oxygen at high temperature, as shown in Figure 14. The macroscopic performance indicated that some coal could not burn or only incomplete combustion occurred, with a decrease in the total heat release of the coal + LMP-Bt sample.

## 3. Conclusions

In summary, a novel, pH-sensitive, sol-gel transition colloid was prepared using low methoxyl pectin (LMP), calcium bentonite (Ca-Bt), sodium bentonite (Na-Bt), and water as the main components. The LMP-Bt colloid featured water retention, thermal stability, no precipitation in sol state, and sol-gel transition by sensing a decrease in pH value. These properties improved the fluidity, leakage sealing, and oxygen isolation effects for coal fire extinguishing.

The resulting critical pH value for the sol-gel transition was 6.5~8.0. The critical pH value for gelation had a larger-the-better characteristic, the optimal levels of the effect of the components on the critical pH value were: 12 g/L LMP, 40 g/L Ca-Bt, and 30 g/L Na-Bt. The Ca-Bt content played a dominant role in the critical pH value because it was the only source of Ca^2+^. The Na-Bt content had no significant effect on the critical pH value. The adhesion rate of LMP-Bt was 65.10%. The use of colloids reduced the specific surface area of coal by 29.37% after the colloid was completely dehydrated. Meanwhile, no exothermic decomposition reaction occurred in the LMP-Bt colloid at 30~900 °C. Adhesion of the LMP-Bt colloid to the coal surface could reduce the risk of coal reignition. This pH-sensitive, sol-gel transition colloid will have potential applications in the field of coal fire extinguishing.

## 4. Materials and Methods

### 4.1. Materials

The materials used in this research are as follows:

(1) LMP (95%, purchased from Henan Weiduomei Biotechnology Co., LTD., Zhengzhou, China)
(2) Ca-Bt (analytically pure, purchased from Tianjin Balance Biotechnology Co., LTD., Tianjin, China)
(3) Na-Bt (purchased from Yanxin Mineral Co., LTD., Shijiazhuang, China)
(4) Acetic acid (HAC, analytically pure, Tianjin Damao Chemical Reagent Factory, Tianjin, China).

### 4.2. Colloid Preparation

**Figure 15:**
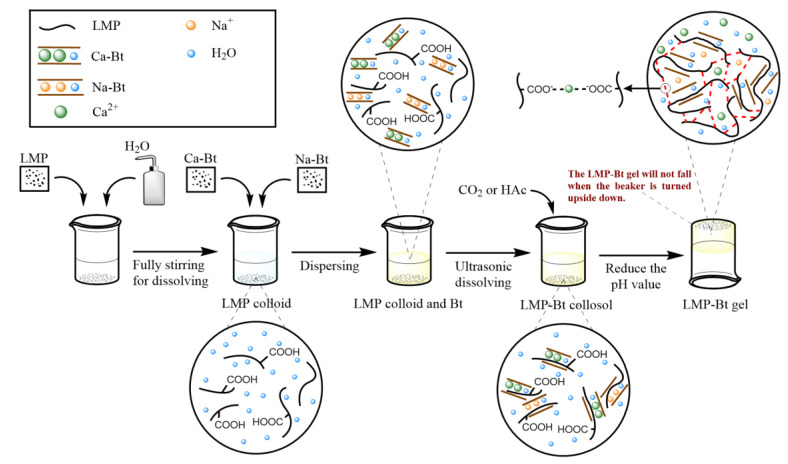
Production process of LMP-Bt colloid.

Figure 15 shows the production process of the LMP-Bt colloid. The detailed process was as follows:

(1) Dissolution of LMP to form collosol: an appropriate amount of LMP powder and 100 mL water were added into the beaker and stirred with an electric stirrer until there were no obvious LMP particles in the beaker.
(2) Addition of Ca-Bt: an appropriate amount of Ca-Bt was added to the collosol and stirred with electric stirrer until there were no obvious Ca-Bt particles in the collosol.
(3) Addition of Na-Bt: Na-Bt was added to the collosol and stirred with an electric stirrer until there were no obvious Na-Bt particles in the collosol.
(4) Ultrasonication: the prepared collosol was put into an ultrasonic dispersion instrument for 30 min, allowing the LMP molecules to be inserted between the Bt layers to form the LMP-Bt collosol.
(5) Validation of the pH sensitivity of the LMP-Bt colloid: acidic gases, such as CO_2_ and SO_2_, or a pre-configured acetic acid solution, were added into the LMP-Bt collosol to reduce the pH value of the collosol. When the pH value of the collosol was lower than the critical pH value for gelation, the collosol turned into the LMP-Bt gel.

### 4.3. Determination of Colloid Microstructures

The LMP-Bt colloid was completely dried at 60 °C and ground to powder. The Ca-Bt powder, Na-Bt powder, and LMP-Bt powder were tested using a D8 Advance X-ray diffractometer (Bruker, Bremen, Germany). The Cu Κ_α_ radiation was designed with a wavelength of 0.154 nm, tube voltages of 20~60 kV (1 kV/1 step), tube currents of 10~60 mA, scanning angles in the range of 4° to 60°, and a scanning rate of 4°/min.

### 4.4. Preliminary Testing

The LMP-Bt colloid featured water retention, thermal stability, no precipitation in sol state, and sol-gel transition by sensing a decrease in pH value. Preliminary experiments of the LMP-Bt colloid with different composition ratios were carried out to determine the reasonable range of each component. The results showed that:

(1) When the LMP content was less than 6 g/L, the sol-gel transition could not be achieved regardless of the content of Ca-Bt. Otherwise, the critical pH for gelation increased with increasing Ca-Bt content. The critical pH for gelation was 6 when the Ca-Bt content was 10 g/L.
(2) A precipitation phenomenon in sol state occurred when the total content of Ca-Bt and Na-Bt was more than 80 g/L. Therefore, the maximums of both the Ca-Bt and Na-Bt contents were set to 40 g/L.
(3) The LMP-Bt colloid samples were put in a water bath at 95 °C. A phenomenon of water draining occurred when the LMP content was 6 g/L. An unexpected sol-gel transition phenomenon occurred when the LMP content was 14 g/L. Therefore, the range of LMP content was set to 8~12 g/L.
(4) The LMP-Bt colloid samples with LMP content between 8 and 12 g/L were put in a water bath at 95 °C. The unexpected sol-gel transition phenomenon occurred when the Ca-Bt content was 10 g/L. Therefore, the range of Ca-Bt content was set to 20~40 g/L. Water draining was the most serious when the LMP content was 8 g/L and the Ca-Bt content was 20 g/L, which could be restrained by increasing the Na-Bt content to 20 g/L. Therefore, the range of Na-Bt content was also set 20~40 g/L.

### 4.5. Performance Testing

#### 4.5.1. pH Sensitivity

##### Orthogonal Experiments to Determine the Critical pH for Gelation

In order to determine the critical pH value of the LMP-Bt colloid for gelation and the effects of the components on the critical pH value, orthogonal experiments were arranged. According to the preliminary testing, the three selected factors (LMP content, Ca-Bt content, and Na-Bt content) and their levels are shown in Table 9. Each factor in these experiments corresponded to 3 levels arranged according to an orthogonal table, as shown in Table 10. Each experiment was repeated twice for a total of 18 experiments.

The test tube inversion experiments were carried out according to the following experimental procedure. First, 0.2% acetic acid solution was added dropwise to the sample, and then the beaker was inverted to observe whether the colloid in the beaker was flowing after stirring. If the colloid adhered and no longer flowed in the beaker, the gel was formed at this time. The colloidal pH value was measured by precision pH test paper and recorded as the critical pH value for gelation of the experimental sample. If the colloid was still flowing, drops of acetic acid solution continued to be added until the sol-gel transition was completed.

##### Experimental Verification of Gelation by Absorbing Gas Products of Coal Combustion

**Figure 16:**
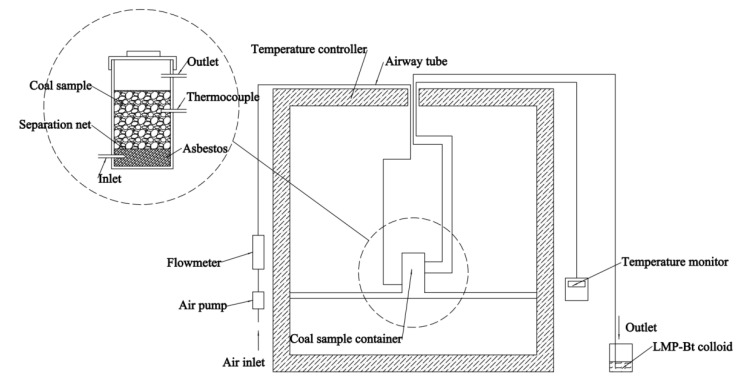
Verification of gelation by absorbing gas products of coal combustion.

In order to verify that the gas products of coal combustion were able to transform the LMP-Bt colloid from collosol to gel, gas products were passed into the LMP-Bt colloid and the flow state of colloid was observed. The experimental procedure is listed as follows:

(1) Coal sample (20 g) with particle size of 2~3 mm was stored in a coal sample container of 42 mm internal diameter and 150 mm height. A 10 mL aliquot of the prepared LMP-Bt colloid was placed in a 50 mL beaker. The experimental device was constructed as shown in Figure 16.
(2) The coal sample was heated using a temperature controller. When the coal temperature (the value of the thermocouple) reached 300 °C, the gas products were passed through the outlet inserted into the LMP-Bt colloid for 5 min.

#### 4.5.2. Rheology

In order to quantitatively characterize the fluidity of the LMP-Bt colloid before and after gelation, and to explore the effect of each component on the rheological properties of the LMP-Bt colloid, single factor experiments with different composition ratios were designed, as shown in Table 11. The Kinexus Pro+ rheometer (Malvern, UK) was used to measure the storage modulus (G’), loss modulus (G’’) and viscosity of the samples. The temperature ranged from 25 to 120 °C and the heating rate was 5 °C/min.

#### 4.5.3. Water Retention

The LMP-Bt colloid sample in Exp-9 was the most sensitive for sol-gel transition and was used in the water retention tests. First, 20 mL LMP-Bt colloid sample was prepared and placed in a 25 mL beaker, followed by stirring and the pH value being adjusted to 6 to achieve gelation. The initial mass of the LMP-Bt gel (*m*_2_) was recorded after standing for 10 min. Then, the LMP-Bt gel was dried at 60 °C in a drying oven. The residual mass of the gel (*m*_1_) was recorded after 12 h. Finally, the water retention rate was calculated by Equation (4).

(4) $$\omega =\frac{m_{1}-m_{s}}{m_{2}-m_{s}}\times 100\%$$

where *ω* is the bulk water retention rate, and *m*_s_ is the mass sum of LMP, Ca-Bt, and Na-Bt.

#### 4.5.4. Oxygen Isolation

The LMP-Bt colloid sample in Exp-9 was used to investigate its oxygen isolation performance. The experimental procedure was as follows:

(1) Sample preparation: the coal was broken into particles with a diameter of 2~3 mm. Two groups of coal samples (10 g) were obtained. One coal sample was placed in a beaker. The LMP-Bt colloid was added into the beaker to cover the upper layer of the coal sample. Then, 0.2% acetic acid solution was added dropwise until the LMP-Bt colloid underwent gelation. The other sample was not processed. Two coal samples were put into the drying oven at 130 °C for 7 h to achieve complete water loss.
(2) Pretreatment: two groups of samples was degassed by vacuum at 100 °C for 1 h.
(3) Physical adsorption test: the physical adsorption instrument ASAP 2460 3.01 (Mack Company, NY, USA) was used to measure the specific surface area of the two groups of samples by the BET method. Here, nitrogen was selected as the adsorbent.

#### 4.5.5. Adhesion

The adhesion performance of the LMP-Bt colloid from Exp-9 was investigated by the following:

(1) Preparation of colloid: the LMP-Bt colloid and sodium carboxymethyl cellulose (CMC-Na) with the same LMP content were prepared for the test.
(2) Weighing of coal samples: six coal samples of the same style were weighed and denoted as *m*_a_.
(3) Adhesion: the pH value of the LMP-Bt colloid was adjusted to 8, and then a critical LMP-Bt colloid that was about to turn into gel was obtained by decreasing the pH value. The coal samples were placed in the critical LMP-Bt colloid/CMC-Na colloid for 30 s. Then, the coal samples were removed from the colloid and kept until no more colloid fell from the surface of the coal. These coal samples were weighed and denoted as *m*_b_. Each test was repeated three times.
(4) Calculation: the adhesion rates of the two kinds of colloids were calculated by the Equation (5).

(5) $$R=\frac{m_{b}-m_{a}}{m_{a}}\times 100\%$$

#### 4.5.6. Thermal Stability

The thermal stability of the LMP-Bt colloid from Exp-9 was investigated by the following:

(1) Sample preparation: the LMP-Bt colloid was mixed with 0.2% acetic acid solution to adjust its pH value to 6 for gelation.
(2) Thermogravimetric differential scanning calorimetry (TG-DSC) experiments were conducted using the STA 449 F5 thermogravimetric analyzer (Netzsch, Bavaria, Germany) with the temperature ranging from 30 to 900 C° and a heating rate of 10℃/min. The test atmosphere was air.

#### 4.5.7. Inhibition

The resilience ability of the LMP-Bt colloid from Exp-9 was investigated by the following:

(1) The coal was broken into particles with a diameter of 2~3 mm. Two groups of coal samples (10 g) were obtained. One coal sample was placed in a beaker. The LMP-Bt colloid was added to the beaker to cover the upper layer of the coal sample. Then, 0.2% acetic acid solution was added dropwise until the LMP-Bt colloid underwent gelation. The other sample was not processed.
(2) TG-DSC experiments were conducted on the two groups of coal samples using the thermogravimetric analyzer with the temperature ranging from 30 to 900 C° and a heating rate of 10 C°/min. The test atmosphere was air.

## Figures and Tables

**Table 1:** Critical pH values for gelation.

	Experiment ID	Exp-1	Exp-2	Exp-3	Exp-4	Exp-5	Exp-6	Exp-7	Exp-8	Exp-9
Group										
First group		6.5	6.5	7.5	6.5	7.0	7.5	6.5	7.5	7.5
Second group		6.5	7.0	7.5	6.5	7.0	7.5	6.5	7.0	8.0

**Table 2:** Values of ${\bar{k}}_{i,j}$ for gelation.

	Factor	LMP Content	Ca-Bt Content	Na-Bt Content
Level				
L-1		6.92	6.50	7.08
L-2		7.00	7.00	7.00
L-3		7.17	7.58	7.00

**Table 3:** Analysis of variance for the critical pH values.

Deviation Point	Quadratic Sum	DOF	Mean Square	*F*	*p*	Significance
LMP content	0.194	2	0.097	2.333	0.153	-
Ca-Bt content	3.528	2	1.764	42.333	0.000	**
Na-Bt content	0.028	2	0.014	0.333	0.725	-
Blank group	0.111	2	0.056	1.333	0.311	-
Residual Error	0.375	9	0.042	-	-	-

**Table 4:** Average viscosity values for Exp-a, Exp-b, and Exp-c.

Experiment ID	Ca-Bt Content, g/L	Average Viscosity, Pa∙s
Exp-a	20	48.16
Exp-b	30	70.03
Exp-c	40	115.01

**Table 5:** Average values of viscosity for Exp-d, Exp-b, and Exp-e.

Experiment ID	LMP Content, g/L	Average Viscosity, Pa∙s
Exp-d	8	53.42
Exp-b	10	70.03
Exp-e	12	52.77

**Table 6:** Water retention rates of LMP-Bt gels.

Experiment ID	ω/%	$\bar{\omega }$/%
Wtr-1	57.99	59.53
Wtr-2	59.75	
Wtr-3	59.64	
Wtr-4	60.49	
Wtr-5	59.77	

**Table 7:** Specific surface areas of coal and coal + LMP-Bt.

Component	Specific Surface Area, $m^{2}/g$
Coal	7.56
Coal + LMP-Bt	5.34

**Table 8:** Results of adhesion tests.

Sample	$m_{1},\;g$	$m_{2},\;g$	R, %	$\bar{R},\;\%$
Sample 1 (Coal + LMP-Bt)	6.51	10.47	60.83	65.10
Sample 2 (Coal + LMP-Bt)	6.52	10.93	67.64	
Sample 3 (Coal + LMP-Bt)	6.36	10.61	66.82	
Sample 4 (Coal + CMC-Na)	6.62	7.62	15.11	16.14
Sample 5 (Coal + CMC-Na)	6.52	7.52	15.34	
Sample 6 (Coal + CMC-Na)	6.45	7.61	17.98	

**Table 9:** Factors and their levels.

	Factor	LMP Content, g/L	Ca-Bt Content, g/L	Na-Bt Content, g/L
Level				
L-1		8	20	20
L-2		10	30	30
L-3		12	40	40

**Table 10:** Orthogonal table.

Experiment ID	LMP Content Level	Ca-Bt Content Level	Na-Bt Content Level
Exp-1	L-1	L-1	L-1
Exp-2	L-1	L-2	L-2
Exp-3	L-1	L-3	L-3
Exp-4	L-2	L-1	L-2
Exp-5	L-2	L-2	L-3
Exp-6	L-2	L-3	L-1
Exp-7	L-3	L-1	L-3
Exp-8	L-3	L-2	L-1
Exp-9	L-3	L-3	L-2

**Table 11:** Single factor experiment settings with different composition ratios.

Experiment ID	LMP Content, g/L	Ca-Bt Content, g/L	Na-Bt Content, g/L	pH
Exp-a	10	20	30	6
Exp-b	10	30	30	6
Exp-c	10	40	30	6
Exp-d	8	30	30	6
Exp-e	12	30	30	6
Exp-f	10	30	30	9

## Data Availability

Data are contained within the article.
